# Supplementary material for: 24-Epibrassinolide Promotes Fatty Acid Accumulation and the Expression of Related Genes in Styrax tonkinensis Seeds
Source: Int J Mol Sci. 2022 Aug 10;23(16):8897. doi: 10.3390/ijms23168897 (PMC9408854; doi:10.3390/ijms23168897)
Supplement: Supplementary file 1 [file ijms-23-08897-s001.zip › Figure S1.pdf]

During seed development, the expressions of *KASII*, *EAR*, *FATB* and *accB* showed a downward trend in general, and the expressions of *KASII*, *FATB* and *accB* in EBL5-treated mother tree seeds were always higher than those in CK seeds. In contrast, the expression levels of *KAR*, *ACSL*, *ACSF3*, *SAD2* and *accA* in seeds of EBL5-treated trees increased first and then decreased. *KAR*, *SAD2* and *accA* were expressed highly at 70 DAF. The expression levels of *ACSL* and *ACSF3* in seeds of EBL5-treated trees reached the maximum at 130 and 100 DAF, respectively. In addition, the expression of *FAD2* in seeds of treated trees was always lower than that of untreated trees during seed development, displaying an 'upward-downward-upward' trend.

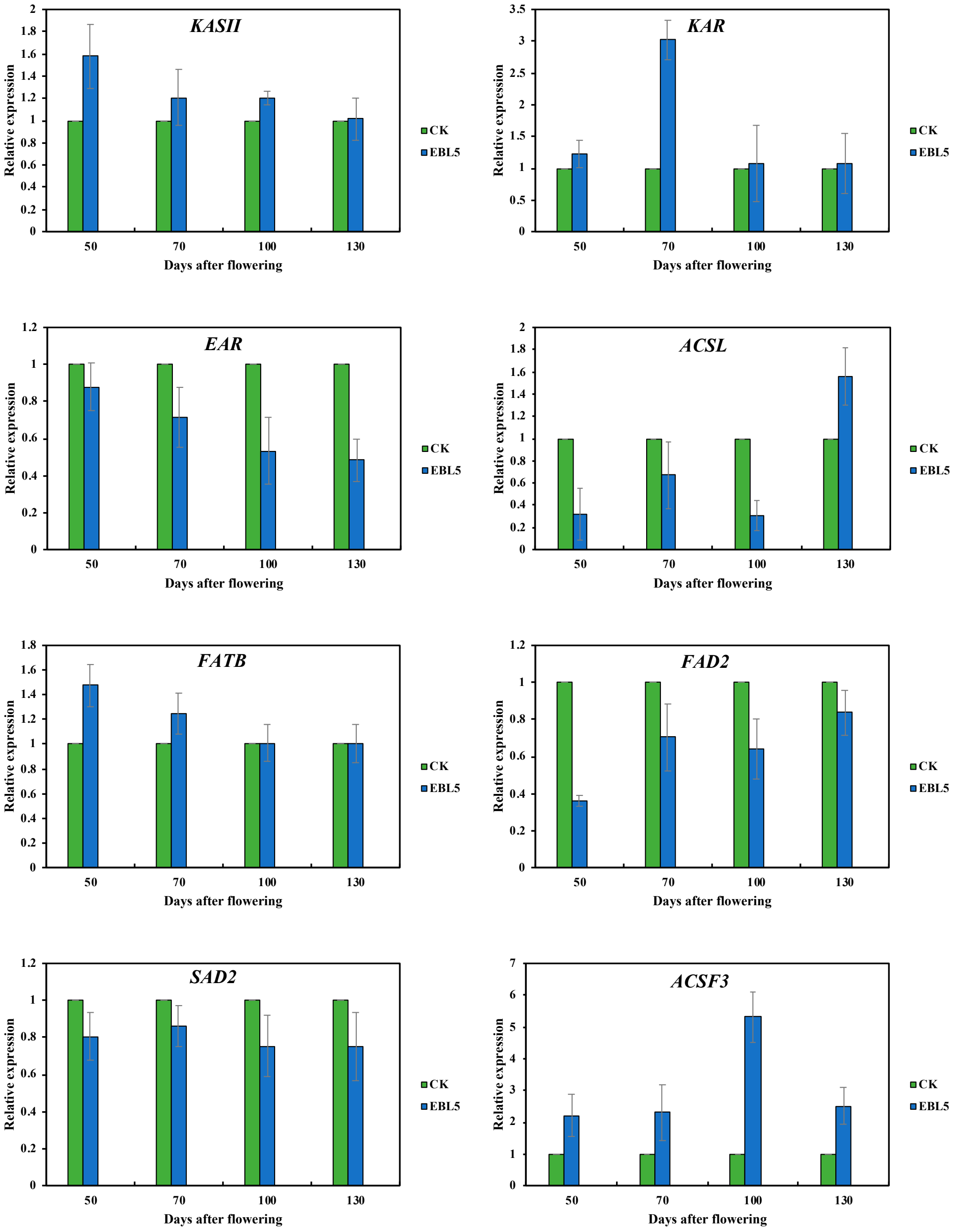

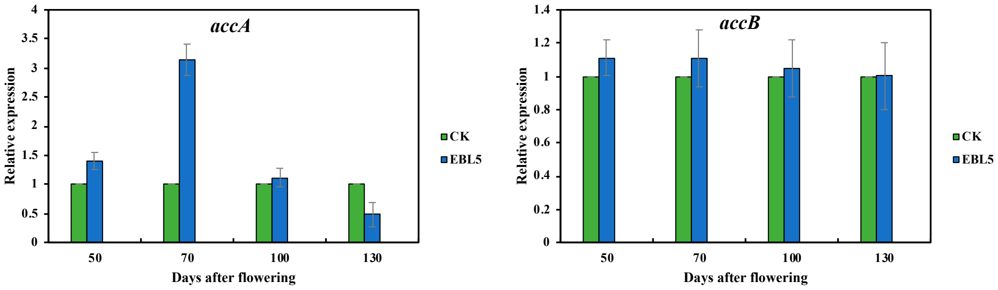

Figure S1. Validation of unigenes expression patterns uncovered by RNA-Seq.
