# Supplementary material for: 24-Epibrassinolide Promotes Fatty Acid Accumulation and the Expression of Related Genes in Styrax tonkinensis Seeds
Source: Int J Mol Sci. 2022 Aug 10;23(16):8897. doi: 10.3390/ijms23168897 (PMC9408854; doi:10.3390/ijms23168897)
Supplement: Supplementary file 1 [file ijms-23-08897-s001.zip › Table S1.pdf]

Table S1. FA compositions in *S. tonkinensis* seeds under EBL5 at four sampling periods.

	FA composition (%)							
	50 DAF		70 DAF		100 DAF		130 DAF	
	CK	EBL5	CK	EBL5	CK	EBL5	CK	EBL5
C14:0	0.17±0.06	0.10±0.05	0.08±0.04	0.02±0.02	0.08±0.05	0.08±0.03	0.08±0.06	0.07±0.03
C15:0	0.10±0.02	0.14±0.02	0.04±0.02	0.02±0.01	0.02±0.01	0.02±0.05	0.02±0.01	0.02±0.01
C16:0	25.53±2.02	24.27±2.12	17.29±3.15	18.35±1.02	11.73±0.94	10.95±1.19	10.41±2.26	10.44±1.31
C16:1	0.24±0.02	0.27±0.05	0.14±0.04	0.19±0.05	0.18±0.01	0.16±0.07	0.13±0.08	0.15±0.05
C17:0	0.64±0.03	0.58±0.03	0.79±0.32	0.72±0.11	0.43±0.16	0.46±0.14	0.37±0.11	0.43±0.08
C17:1	0.41±0.03	0.34±0.02	0.37±0.18	0.36±0.07	0.31±0.10	0.31±0.02	0.24±0.06	0.25±0.05
C18:0	5.95±0.78	5.26±0.47	3.17±0.74	3.18±0.35	2.58±1.27	3.18±1.89	3.79±1.57	3.65±0.67
C18:1	13.80±1.30	15.69±1.56	11.65±2.06	11.72±1.12	23.33±2.65	25.14±3.17	29.36±2.00	27.00±1.50
C18:2	24.86±1.70	26.18±1.78	53.86±5.04	51.98±4.43	53.43±6.00	50.59±6.57	44.25±7.00	46.89±5.50
C18:3	18.63±1.85	18.01±0.99	8.52±1.50	9.29±0.68	6.89±1.17	7.64±1.84	9.99±2.62	9.72±1.17
C20:0	2.30±0.22	2.26±0.62	0.85±0.16	0.82±0.07	0.24±0.14	0.38±0.03	0.38±0.13	0.41±0.05
C20:1	1.45±0.25	1.16±0.20	0.61±0.24	0.51±0.08	0.24±0.08	0.29±0.04	0.37±0.18	0.31±0.15
C20:2	0.41±0.01	0.44±0.02	0.22±0.01	0.31±0.02	0.06±0.03	0.08±0.03	0.05±0.01	0.07±0.01
C20:3	0.37±0.01	0.38±0.01	0.20±0.01	0.24±0.01	0.03±0.01	0.05±0.02	0.02±0.02	0.03±0.01
C21:0	0.61±0.02	0.58±0.03	0.30±0.04	0.31±0.01	0.04±0.02	0.07±0.03	0.04±0.01	0.05±0.01
C22:0	1.72±0.22	1.74±0.14	0.65±0.11	0.65±0.04	0.16±0.01	0.22±0.11	0.20±0.03	0.23±0.02
C22:1	0.17±0.06	ND	ND	ND	ND	ND	0.02±0.01	0.01±0.01
C23:0	0.54±0.02	0.55±0.02	0.28±0.03	0.31±0.01	0.06±0.03	0.09±0.04	0.06±0.01	0.07±0.04
C24:0	1.62±0.16	1.57±0.14	0.71±0.11	0.70±0.05	0.14±0.10	0.23±0.13	0.17±0.08	0.19±0.02
C24:1	0.47±0.01	0.48±0.01	0.28±0.02	0.31±0.02	0.06±0.04	0.06±0.01	0.04±0.02	0.03±0.01

Note: ND indicated not detected. C14:0, tetradecanoic acid; C15:0, pentadecanoic acid; C16:0, palmitic acid; C16:1, palmitoleic acid;

C17:0, heptadecanoic acid; C17:1, ginkgolic acid; C18:0, stearic acid; C18:1, oleic acid; C18:2, linoleic acid; C18:3, linolenic acid; C20:0, eicosanoic acid; C20:1, cis-11-eicosenic acid; C20:2, cis-11,14-eicosadienoic acid; C20:3, cis-11,14,17-eicosatrienoic acid; C21:0, n-heneicosanoic acid; C22:0, docosanoic acid; C22:1, erucic acid; C23:0, tricosanoic acid; C24:0, lignoceric acid; C24:1, nervonic acid.
