# Supplementary material for: 24-Epibrassinolide Promotes Fatty Acid Accumulation and the Expression of Related Genes in Styrax tonkinensis Seeds
Source: Int J Mol Sci. 2022 Aug 10;23(16):8897. doi: 10.3390/ijms23168897 (PMC9408854; doi:10.3390/ijms23168897)
Supplement: Supplementary file 1 [file ijms-23-08897-s001.zip › Table S4.pdf]

Table S4. Primers used in this experiment.

Primer	Sequence
ACSL-F	TTGTTGGTGGATTCAGTG
ACSL-R	ATGCCTTTCTCATCAACCTG
FAD2-F	AGCCGCCCTTCACTGTTG
FAD2-R	GCGTTGCCATACTTCTCG
EAR-F	TGCTATCCAAAGCCAGTC
EAR-R	CAAATGCTCCTATCCAGAA
SAD2-F	CCTCTACTTTCCACCTACC
SAD2-R	CAGCCCACTTGATGTATG
KAR-F	TGCCAACCAAACCAACAA
KAR-R	ACTCGGGATGGGCTGATG
FATB-F	GTTCTTCCCTTCACAATC
FATB-R	AGTCCCTCACTGCTGTAT
ACSF3-F	TGCTGGAATACTAGGGACT
ACSF3-R	CATAGAAAGGGACAAGAAAA
KASII-F	GTAAATGGGATGAGGGAC
KASII-R	CAAACTTATGGCATAGGAA
accA-F	AGTCTTTCCTGCCTTTGC
accA-R	TTGAGCCACCTTCGTTCT
accB-F	TGCTCCTGTCGTTATGTT
accB-R	TAAGAGGTGGAAGGGATG
18s-F	AGTATGGTCGCAAGGCTGAAA
18s-R	CAGACAAATCGCTCCACCAA
